# Minimal impacts of invasive *Scaevola taccada* on *Scaevola plumieri* via pollinator competition in Puerto Rico

**DOI:** 10.3389/fpls.2024.1281797

**Published:** 2024-01-25

**Authors:** Susan M. Swensen, Adriana Morales Gomez, Colette Piasecki-Masters, Ngawang Chime, Abigail R. Wine, Nandadevi Cortes Rodriguez, James Conklin, Peter J. Melcher

**Affiliations:** Department of Biology, Ithaca College, Ithaca, NY, United States

**Keywords:** *Scaevola plumieri*, *Scaevola taccada*, Goodeniaceae, pollination, invasive

## Abstract

**Introduction:**

*Scaevola taccada* and *Scaevola plumieri* co-occur on shorelines of the Caribbean. *Scaevola taccada* is introduced in this habitat and directly competes with native dune vegetation, including *S. plumieri*, a species listed as locally endangered and threatened in Caribbean locations. This study addresses whether the invasive *S. taccada* also impacts the native *S. plumieri* indirectly by competing for pollinators and represents the first comparative study of insect visitation between these species.

**Methods:**

Insect visitation rates were measured at sites where species co-occur and where only the native occurs. Where species cooccur, insect visitors were captured, identified and analyzed for the pollen they carry. Pollen found on open-pollinated flowers was analyzed to assess pollen movement between the two species. We also compared floral nectar from each species by measuring volume, sugar content, and presence and proportions of amine group containing constituents (AGCCs).

**Results:**

Our results demonstrate that both species share insect visitors providing the context for possible pollinator competition, yet significant differences in visitation frequency were not found. We found evidence of asymmetrical heterospecific pollen deposition in the native species, suggesting a possible reproductive impact. Insect visitation rates for the native were not significantly different between invaded and uninvaded sites, suggesting that the invasive *S. taccada* does not limit pollinator visits to *S. plumieri*. Comparisons of nectar rewards from the invasive and the native reveal similar volumes and sugar concentrations, but significant differences in some amine group containing constituents that may enhance pollinator attraction.

**Conclusion:**

Our analysis finds no evidence for pollination competition and therefore *S. taccada*’s main impacts on *S. plumieri* are through competitive displacement and possibly through reproductive impacts as a consequence of heterospecific pollen deposition.

## Introduction

Invasive plants can negatively impact native plants and their communities in a variety of ways. Two primary modes of impact include direct competition for resources and indirect influences through ecosystem modification (Levine et al., 2003; Gioria and Osborne, 2014; Carboni et al., 2021). Competitive displacement via direct competition for water, nutrients, light, and space is a well-documented explanation for the loss of native species in invaded habitats (Levine et al., 2003), however; indirect influences through ecosystem modification may also exist and may be more difficult to detect (Hulme et al., 2013; Jarić et al., 2019). Indirect impacts of invasive plant species may include changes in soil chemistry (Weidenhamer and Calloway, 2010; Santoro et al., 2011; Suseela et al., 2016), nutrient cycling (Ehrenfeld, 2003; Liao et al., 2008; allelopathy (Calloway and Ridenour, 2004), and disturbance regimes (Mack and D’Antonio, 1998; Brooks et al., 2004).

In addition, because invasive plants are often generalists, their integration into animal-based pollination and dispersal mutualisms is enhanced (Richardson et al., 2000; Traveset and Richardson, 2014) and may lead to alterations in the abundance and behavior of such mutualists (Traveset and Richardson, 2006; Parra-Tabla and Arceo-Gómez, 2021). Invasive plants may disrupt native plant-pollinator relationships by affecting pollinator populations (e.g. expanding populations by adding resources to the environment) or by affecting pollinator behavior (e.g. changing foraging patterns). If invasive and native species share pollinators, the presence of an invasive could be facilitative (positive), competitive (negative) or neutral in its effect on the native (Rathcke, 1983; Bjerknes et al., 2007). In facilitation, the alien species increases the visitation rate of pollinators to the native species, potentially increasing reproductive success (Thomson, 1978; Ghazoul, 2006; Braun and Lortie, 2019). Conversely, the invasive may compete for pollinators, causing a decrease in visitation rates to native species and a possible decrease in reproductive success (Mitchell et al., 2009). Competition for pollinators resulting negative reproductive outcomes for native species has been described for purple loosestrife (Brown and Mitchell, 2001; Brown et al., 2002) and leafy spurge (Larson et al., 2006) as examples. Invasives that share pollinators with natives may also exert detrimental effects through the deposition of heterospecific pollen onto native stigmas which may result in stigma clogging, inhibition of fertilization by chemical interference, or by the formation of invasive-native hybrids depending on the phylogenetic relatedness of the invasive to the native (Brown and Mitchell, 2001; Streher et al., 2020). Facilitation between invasive alien species and native species has been described for *Carpobrotus* spp. (Moragues and Traveset, 2005; Bartomeus et al., 2008) and between two invasives (*Carduus pycnocephalus* and *Lupinus arboreus*; Molina-Montenegro et al., 2008), however; instances of invasive species facilitating the pollination of native species appear to be infrequent. Most examples of facilitation have been reported among co-occurring native species (Laverty, 1992; Geer et al., 1995; Moeller, 2004; Ghazoul, 2006).

Here, we consider the potential impact of *Scaevola taccada* (beach naupaka) on *Scaevola plumieri* in Puerto Rico. This species pair represents an example of an invasive species (*S. taccada*) interacting with a native congener and enables the opportunity to explore whether this invasive exerts both direct and indirect impacts on the native. Specifically, we focus on indirect impacts by characterizing nectar rewards, insect visitation rates in invaded and uninvaded localities, and pollen movement between the species.

**Figure 1:**
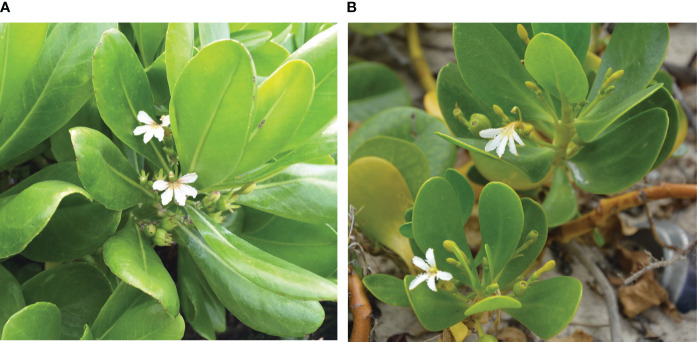
Floral morphology of **(A)** *Scaevola taccada* and **(B)** *Scaevola plumieri*. The stylar indusium (a synapomorphy for Goodeniaceae) is located above the half-flower in *Scaevola* species. The indusium grows up through mature anthers and secondarily presents the pollen to pollinators, then later become a receptive stigmatic surface. At our study locations, we frequently observed flowers of *S. taccada* to have purple streaks not observed in *S. plumieri*; corolla lobes of *S. plumieri* appear fringed, a morphology not observed in *S. taccada*.

The genus *Scaevola* (Goodeniaceae) comprises 130 species, the majority of which occur only in Australia. *Scaevola* (and Goodeniaceae, more generally) are distinguished by a unique floral character, the stylar indusium (**Figure 1**; Leins and Erbar, 2006). *Scaevol*a’s characteristic half-flowers are borne in cymes arising from upper leaf axils. *Scaevola* are protandrous, with pollen secondarily presented by the stylar indusium (Leins and Erbar, 2006).

Approximately 40 species of *Scaevola* exist outside of Australia, the majority of which are narrow endemics of Pacific islands (Patterson, 1995; Howarth et al., 2003). *Scaevola plumieri* (L.) Vahl. and *Scaevola taccada* (Gaertn.) Roxb. (= *S. sericea* Vahl) (**Figure 1**) are pantropical. *S. plumieri* has an Indo-Atlantic distribution (Espejel, 1987; Peter et al., 2003; Knevel and Lubke, 2004) while *S. taccada* is natively Indo-Pacific (Howarth et al., 2003). Both species have fleshy fruits that can remain viable in salt water for months (Guppy, 1917; Lesko and Walker, 1969) and are also eaten and potentially transported by birds (Carlquist, 1969). In their native ranges, these species are common and significant components of strand vegetation where they build and stabilize dunes (Peter et al., 2003). Both are perennial shrubs capable of vegetative and sexual reproduction.

*Scaevola taccada*’s distribution has expanded through escape from cultivation and subsequent naturalization in many areas including the Caribbean (Eshbaugh and Wilson, 1985; Grande and Nozawa, 2010; Finkle and Elliot, 2011; Acevedo-Rodriguez and Strong, 2012; Lockhart, 2012). It was introduced to Florida in the 1960s for landscaping and control of beach erosion (Kaufman and Kaufman, 2007; CABI, 2023). It is now listed as a Category 1 invasive exotic in Florida (FLEPPC, 2019) and is considered an agricultural and environmental weed elsewhere (Randall, 2012). In the Caribbean, *S. taccada* can form large, dense stands displacing native dune vegetation and also possibly preventing sea turtle access to nesting areas (Burton, 2008; CABI, 2023). Displaced native plants include *S. plumieri*, a smaller and less compact shrub. In the Cayman Islands, *S. plumieri* is critically endangered (DaCosta-Cottam et al., 2009) largely due to competition by *S. taccada* and *Casuarina equisitifolia*. *S. plumieri* is listed as a threatened species in Florida (Florida Administrative Code, 2020) and Castillo-Campos et al., 2021; Castillo-Campos et al., 2022 describe the displacement of *S. plumieri* by *S. taccada* in Cozumel.

Because *Scaevola taccada* and *S. plumieri* share similar flowering phenology, flower morphology, and habitat preference, it is likely that they also share similar suites of pollinators, providing the opportunity for the invasive to indirectly impact its congeneric counterpart through competition for pollination (Brown and Mitchell, 2001; Vanparys et al., 2008; Powell et al., 2011). In its native range, *S. taccada* is visited and pollinated primarily by bees, particularly bees in the genus *Apis*, and to a lesser degree, wasps. (Ellmore, 2008; Liao, 2008; Aluri et al., 2019). *S. taccada* plants are typically larger than the native and produce many more flowers. Due to its larger floral resource, we expect that *S. taccada* would exert significant indirect effects on S. *plumieri* through competition with pollinators.

To assess the potential indirect impacts of the *S. taccada*, we studied the pollination biology of *S. taccada* and *S. plumieri* in Puerto Rico where these species co-occur. The goals of our study were to 1) characterize and quantify the floral visitors to both species; 2) analyze the pollen carried by insect visitors to *Scaevola* and; 3) characterize and compare the nectar rewards offered by each species. This represents the first comparative study of insect visitation between these species. By investigating these aspects of pollination biology, we aim to assess the risk that *S. taccada* may pose to the reproductive success of the native *S. plumieri*.

## Materials and methods

### Floral visitation observation

**Figure 2:**
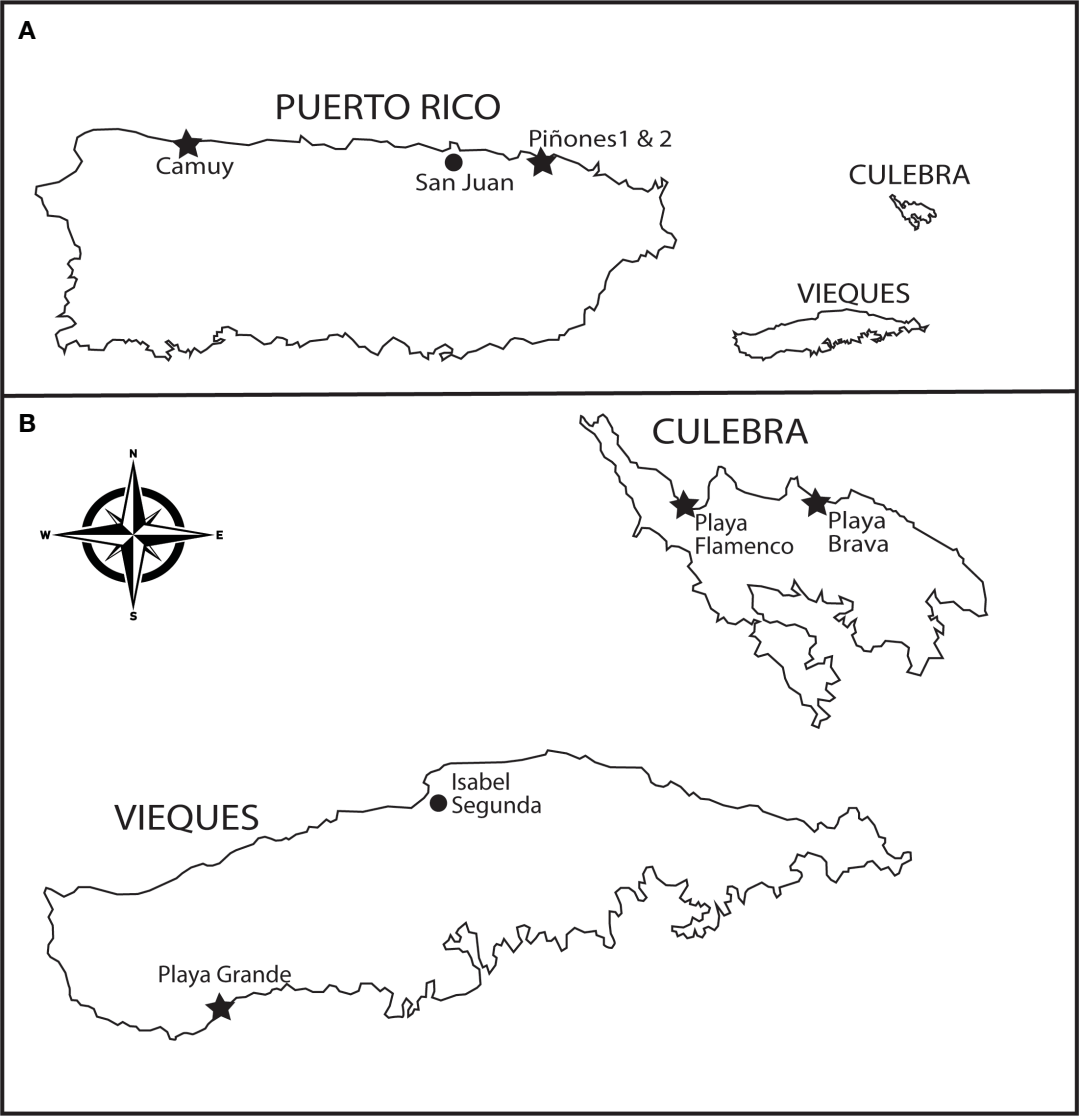
Puerto Rico sampling and observation sites. **(A)** Study locations (stars) on the main island include Camuy (18.489145, -66.845474), Piñones 1 (18.450074, -65.951980), and Piñones 2 (18.446056, -65.933833). **(B)** Study locations on smaller islands (island sizes not to scale) include the Vieques study site at Playa Grande (18.08847 -65.51550) and the Culebra collection sites at Playa Flamenco (18.328483 -65.316855) and Playa Brava (18.329939, -65.284600).

Observations of insect visitation to both species of *Scaevola* took place across three years (2015-2017) at Playa Grande (Vieques Island, Puerto Rico; **Figure 2B**). At the Playa Grande site, both species were distributed every 50-100 m along a sandy foredune on the southeastern shoreline of Vieques. Observations took place at one large individual of *Scaevola taccada* and at two individuals of *S. plumieri* located approximately 75 and 150 m away. These plants were observed during the same week in January over three consecutive years, and on sunny days. Multiple 15-min observation periods were recorded by observers standing 2-3 m away and downwind from plants. Observers recorded the number of flowers under observation for each 15 min period. Observations were followed by capture of insect visitors for later identification and analysis. Each visitation observation documented the identity of the visitor, the number of flowers visited, and the length of visit time per flower. Our observation times coincided with peak insect visitation times (0900-1500) and our observation times totaled approximately 20 hours spread across three years. Sample sizes are shown in **Table 1**. Differences in visitation frequency between *S. taccada* and *S. plumieri* were tested for significance in each of the three years and with all years combined.

**Table 1:** Percentages of insect visitors to *Scaevola* in Playa Grande*(Vieques) and Camuy, Puerto Rico.

Visits (as % of total)		2015 (PG)		2016 (PG)		2017(PG)		2020 (Camuy)
^1^Visiting Species		*S. plumieri* *(6)*	*S. taccada (2)*	*S. plumieri (19)*	*S. taccada (21)*	*S. plumieri (18)*	*S. taccada (12)*	*S. plumieri* *(32)*
Bees								
*Apis mellifera*		1.22	2.25	15.70	20.14	32.11	97.62	64.9
*Centris decolorata*		35.98	96.63	2.48	49.28	−	1.08	5.0
*Centris lanipes*		−	−	−	18.71	−	−	−
*^2^Lasioglossum* sp.		3.05	−	18.18	1.80	−	−	−
*Megachile concinna*		−	−	52.89	9.35	−	−	−
*Xylocopa mordax*		−	−	−	0.72	−	−	−
*Unidentified bee*		−	−	−	−	−	−	0.7
Wasps								
^3^ *Campsomeris* sp.		19.51	−	−	−	66.32	1.08	19.8
*Prionyx thomae*		35.59	−	−	−	−	−	−
*^4^Stictia* sp.		−	−	6.61	−	1.57	0.22	2.2
*Unidentified wasp*		−	−	−	−	−	−	2.0
Butterflies								
*Agraulis* sp.		3.05	1.12	1.65	−	−	−	−
*Ascia monsute*		0.61	−	2.48	−	−	−	−
*Panoquina* sp.		−	−	−	−	−	−	5.4

*In addition to these observed visitors, the bee *Exomalopsis analis* was captured (but not observed to visit flowers) in 2015 at Playa Grande. ^1^Named species confirmed by capture. ^2^Caught specimens included both *Lasioglossum dispersum* and *L. enatum* (J. Gibbs, personal communication). ^3^Includes *Campsomeris trifasciata* and *Campsomeris dorsata* (caught at Playa Grande in 2015 and at Camuy). ^4^Includes *Stictia signata* (caught at Playa Grande) and an unidentified *Stictia* species observed at Camuy. For each year, the most common visitors to flowers are listed by percentage of the total visits. Dashes indicate the visiting species was not observed in that year. Sample sizes (the number of independent 15 min observation periods) are included for each year and plant species.

Observations of insect visitation to *S. plumieri* were made in January 2020 at three additional locations on the main island of Puerto Rico (Camuy, Piñones 1, Piñones 2; **Figure 2A**). The Camuy site is uninvaded (no *S. taccada* present) and located on the north shore near the town of Camuy. Here, *Scaevola plumieri* is abundant for more than 1500 m along the shoreline with individuals occurring every 10-50 m. We observed flower visitation at five different locations for a total of 465 min over two days. At the Camuy site, we captured and identified insect visitors. Piñones 1 and 2 are both invaded sites along the north shore east of San Juan where both *Scaevola* species occur every 25-200 m. At Piñones 1, we observed *S. plumieri* on a single day at four locations for a total of 195 min. At Pinones 2 we observed on a single day at five locations for a total of 615 min. Single day observations were a result of travel constraints due to earthquakes in Puerto Rico in 2020. We did not capture insects at the Piñones sites. Visitation observations were carried out as described for those at Playa Grande. Comparisons of visitation rates for *S. plumieri* at invaded vs. uninvaded sites were tested for significant differences.

### Statistical analysis

Visitation data was assessed for normality by calculating skewness and kurtosis values and by visually inspecting histograms and Q-Q plots using Microsoft Excel (Mac version 16.71; Microsoft Corporation 2023). Because not all data were normally distributed, we log-transformed our data before conducting a two sample T-Test (Microsoft Excel) to look for significant differences in visitation frequency between species in each year (and all years combined) and for visitation frequency for *S. plumieri* at invaded vs. uninvaded sites. Data transformation improved the normality of most data, but not all. We also conducted Mann Whitney U tests (in Microsoft Excel) with non-transformed data to look for significant differences, as our sample sizes for 2015 were low (see **Table 1**). LMM (linear mixed model) analysis was also performed using plant species as fixed effect and the six different observations (two species in each of three years) as random effect. Analysis was performed using the Ime4 package (version 1.1-35.1, Bates et al., 2015) in R (R Core Team, 2023). In each case, our statistical tests revealed no significant differences in visitation frequency and in most cases we report only the results from the Mann-Whitney U tests.

### Floral pollen analysis

Anthers from unopened *Scaevola* flowers were dissected and mounted onto slides in fuchsin jelly (Beattie, 1971) to establish reference slides for each species’ pollen. Pollen was photographed using a Nikon Eclipse E800 microscope (Melville, NY, USA) with NIS-Elements imaging software (version 4, 2013). Pollen from images was measured using ImageJ (version 1.51; Schneider et al., 2012). Because of the morphological similarity of the pollen from *S. taccada* and *S. plumieri*, we built frequency distributions of pollen sizes for each species. Over 200 pollen grains from each species were characterized. Pollen was taken from unopened flowers from seven different *S. plumieri* individuals and nine different *S. taccada* individuals.

Open-pollinated flowers were also collected in each year of our study at Playa Grande on the island of Vieques (**Figure 2B**). In each year, we collected 15 flowers per species (from 2-3 individuals per species) and stored them separately in 70% isopropanol for subsequent analysis. These flowers were dissected, and pollen grains found attached to the stylar indusia were mounted and measured as described above. Because the size class distribution of pollen diameters of the two species overlapped, we employed a rule to classify pollen from each species: pollen diameters >37.73 µm were assigned to *S. taccada* whereas diameters <32.95 µm were assigned to *S. plumieri*. This rule is conservative in that it avoids assigning identity to any pollen grains in the size range where the distributions overlap.

Pollen surfaces were compared using field emission scanning electron microscopy (Zheng et al., 2020) at the Cornell University SEM facility (LEO 1550 Keck FE-SEM; Cambridge, MA, USA). Pollen was collected from unopened flowers collected at Playa Grande, Vieques in 2017 and stored in 70% isopropanol. Pollen samples were prepared by adsorption onto a silicon wafer which was attached to a flat aluminum platform sample holder and coated with gold palladium for a duration of 20 s at a current of 10 mA. FE-SEM images were obtained with the microscope operating at 1.00 kV and at a working distance of 5 mm with an aperture size of 30 µm.

### Insect capture and pollen analysis

Insects visiting either *S. plumieri* or *S. taccada* at Playa Grande were captured using a butterfly net and transferred into small jars containing plaster of Paris (DAP, Baltimore, MD, USA) saturated with ethyl acetate (non-acetone fingernail polish remover). Pollen adhering to dorsal and ventral body surfaces and appendages was removed by swabbing with a ~5mm square of fuchsin jelly (Beattie, 1971). The jelly was then melted onto a microscope slide under low heat before a coverslip was placed on top. After cooling, coverslips were sealed with clear fingernail polish. For short-term storage after removing pollen, insects were submerged in 70% ethanol. Later, insects were dried and mounted for identification. Pollen swipes were viewed using light microscopy (as described for flower pollen above). Pollen grains resembling *Scaevola* were identified, measured, and assigned to species as described above.

### Nectar collection and analysis

Flowers of both species were bagged 1-2 days prior to nectar collection using fine mesh bags and petroleum jelly was applied to stems below the bags to deter ants from stealing nectar. Nectar was collected using 1-5µL graduated micro-capillary tubes (Drummond Scientific) and the volume per flower was recorded. We avoided collecting nectar just after rainfall or heavy dew, waiting instead to make collections when plants were dry. Nectar volume and sugar content was measured in each year (2015-2017) from the Playa Grande location from two *S. plumieri* individuals and two *S. taccada* individuals. For nectar volume measurements, multiple flowers ranging from 10-44 flowers per individual were measured. Nectar concentration (g sugar per 100 g solute or % w/w) was measured from pooled samples with a MISCO Digital refractometer (PA201; Solon, OH, USA). Samples were diluted with 5 µL of RO H_2_O if the combined sample volumes were less than 3 µL. After field measurements, nectar was collected in micro centrifuge tubes and refrigerated for short term storage, then frozen for long-term storage until amino acid analysis. To compare nectar volumes and sugar concentration among species and years, we used a Mann Whitney U test.

### Analysis of amine-group containing constituents in nectar

Amine-Group Containing Constituents (AGCCs) were analyzed in samples collected from two localities on the island of Culebra (**Figure 2B**) in 2016. These collections included 11 samples of *S. plumieri* nectar from 6 different individuals (3 from Playa Brava and 3 from Playa Flamenco; **Figure 2B**) and 6 samples of *S. taccada* nectar from 4 different individuals (2 from Playa Brava and 2 from Playa Flamenco). To assess the presence and concentration of AGCCs, the 17 samples were derivatized following AccQtag protocol (Waters Corporation, Milford, MA, USA). HPLC analysis was performed using a Waters Alliance 2690 HPLC system. Blanks were scanned at the beginning of each run to ensure proper calibration. The binary solvent system prepared for the HPLC included 1:9 AccQTag-dH20 mix and 6:4 acetonitrile-water mix (Parchem, New Rochelle, NY, USA). The samples were extracted with buffer, taking into account if the samples were previously diluted with water. 10 µL of extracted sample was loaded into the HPLC using a Nova-Pak C18 column (4.6 mm X 150 mm). Samples were detected with a Waters model 2475 multi wavelength fluorescent detector with an excitation wavelength of 250 nm and an emission wavelength of 395 nm. Data were collected using Empower 3 (Waters Corp.). AGCCs were identified by comparing the retention times of the samples to the retention times of amino acid standards and the internal standard, norleucine (Sigma-Aldrich, St. Louis, MO, USA). To determine concentration of individual constituents, peak areas were compared to standard curves prepared using the known amino acid standards. Individual AGCC concentrations were added to calculate total concentration. From these data, we then determined the proportion of each AGCC to the total.

A Pearson correlation coefficient was calculated using IBM SPSS Statistics for Windows (version 25; 2017) to determine intraspecific variation in AGCC concentration and composition. Intraspecies coefficients of variation were calculated for concentration and composition separately by dividing the standard deviation by the mean for each constituent. The Wilcoxon rank sum test was implemented using SPSS and (R Core Team, 2023) to determine the interspecies variation since variability in the total concentration and composition of constituents presented outliers. This test is a non-parametric alternative to the t-test and is less sensitive to outliers and non-normality.

## Results

### *Scaevola* insect visitation observation

**Figure 3:**
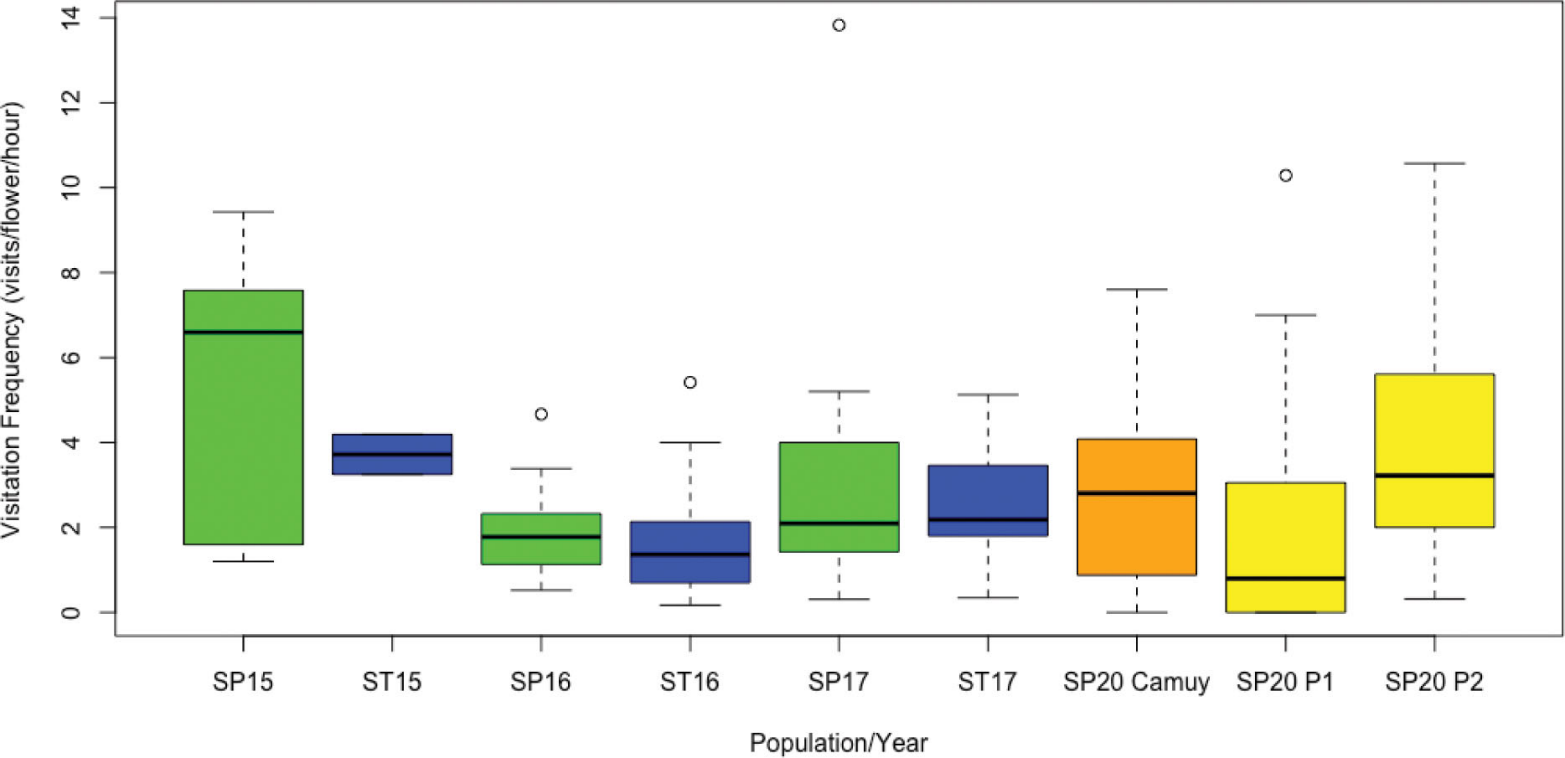
Comparison of overall insect visitation frequency for *Scaevola plumieri* (SP) and *S. taccada* (ST). Insect visitation frequencies are shown for *S. plumieri* (green boxplots) and *S. taccada* (blue boxplots) at the invaded site (Playa Grande) for 2015-2017 and for *S. plumieri* at the uninvaded site (Camuy, orange boxplot) and two invaded sites (P1 and P2; yellow boxplots) for 2020. Lines inside the boxplot represent medians, the bars above and below represent maximum and minimum values, and circles indicate outliers. Mann Whitney U tests revealed no significant differences between species in any particular year, nor between species all years combined, nor between *S. plumieri* at invaded sites (Playa Grande) vs. uninvaded sites (Camuy). Plot produced in R (R Core Team, 2023).

Over three years and approximately 20 hours of observation time at the Vieques (Playa Grande) study site, we identified eight different species of bees, four species of wasps, one hummingbird (Antillean Crested Hummingbird, *Orthorhyncus cristatus*), and two species of butterfly (*Agraulis* sp. and *Ascia monsute*) visiting *Scaevola* plants (**Table 1**). Although the frequency and identity of these visitors varied from year to year, nearly all species were observed to visit both the invasive *S. taccada* and the native *S. plumieri* during our study. One exception was *Lasioglossum* spp. which we observed visiting *S. plumieri* in 2015 and 2016 but did not observe visiting *S. taccada*. Comparison of visitation rates between species in each year (and between species in years combined) revealed no significant differences (Mann Whitney U test; p values ranged from 0.07 to 0.82; **Figure 3**). LMM analysis of visitation rates also did not find significant differences between species in any year.

At Playa Grande, we observed that smaller bees (*Lasioglossum, Megachile*) often did not touch the indusium when entering the flower, and therefore are likely not effective pollinators compared to larger bees (*Centris*, *Xylocopa*) and wasps (*Campsomeris*). We also observed a nectar robbing behavior of *A. mellifera* who occasionally approached the flowers (of both species) from the side, preventing contact with the indusium above.

For comparative purposes, we observed insect visitation for *Scaevola plumieri* at an uninvaded locality on the main island of Puerto Rico in 2020 (Camuy; **Figure 2A**). We identified similar species visiting *S. plumieri* at this site (**Table 1**) but observed *Apis mellifera* to be a more dominant visitor. Comparisons of visitation rates for *S. plumieri* between Playa Grande (2015-2017) and Camuy (2020) revealed no significant differences (Mann Whitney U test; p = 0.44; **Figure 3**). We also compared visitation rates between Camuy and two other invaded locations on the main island in 2020 (Piñones1 and Piñones2; see **Figure 2**). We saw no significant differences in visitation rates between Camuy and these two invaded localities in that year (p = 0.12 and p = 0.07).

A common visitor at all sites was *Apis mellifera*, the European honeybee, an introduced species in Puerto Rico. We were curious whether we might see higher visitation by *A. mellifera* at invaded sites compared to the uninvaded site at Camuy, but we found the opposite. *Apis mellifera* visitation at Camuy was significantly higher (average rate = 1.9 visits/flower/hour) than at Playa Grande (average rate = 0.42 visits/flower/hour; p value <0.00001), though we saw no significant differences between Camuy and the other invaded sites on Puerto Rico in that year (Piñones1 and Piñones2; p = 0.65 and p = 0.20).

### *Scaevola* pollen analyses

**Figure 4:**
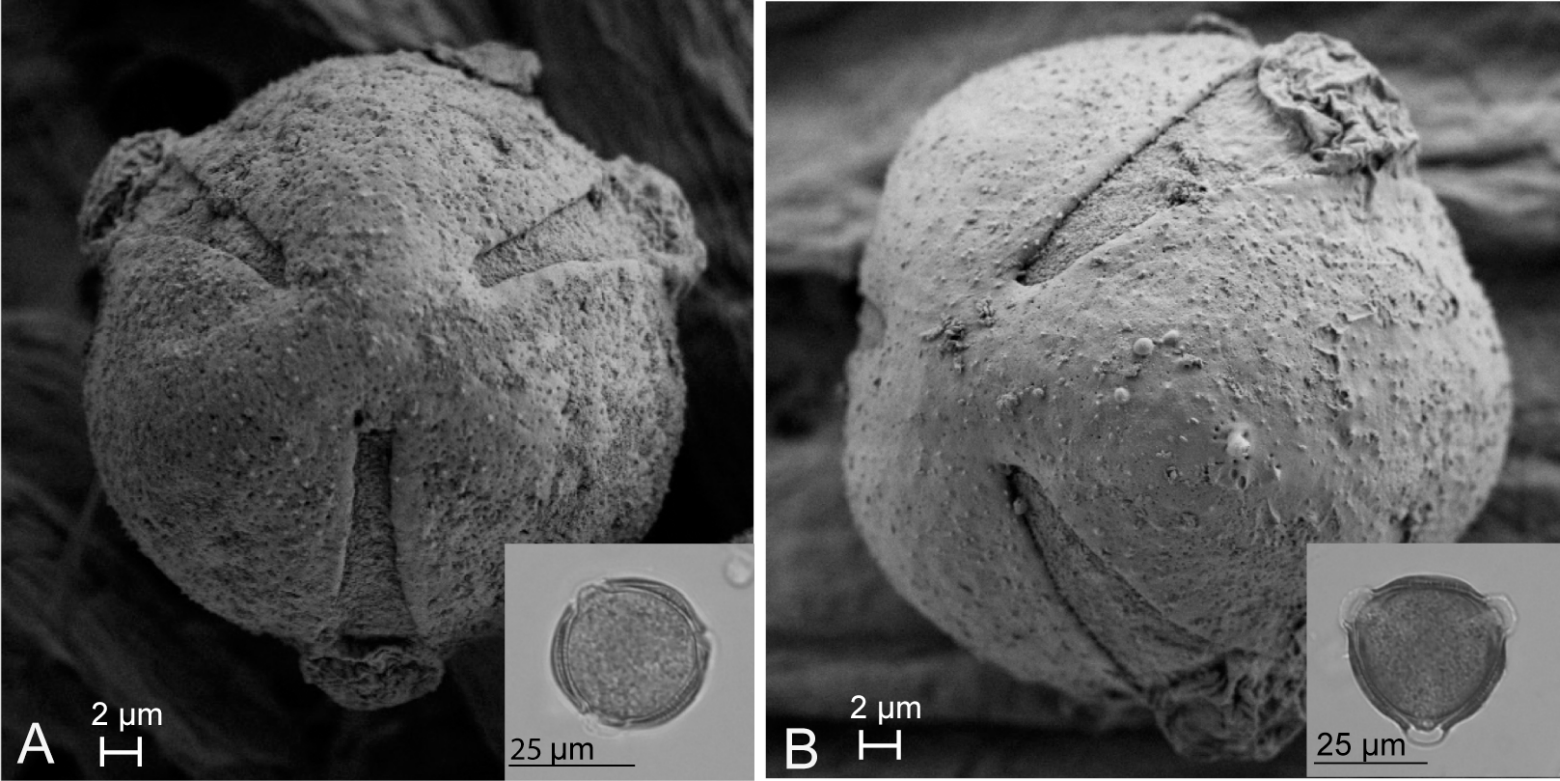
FE-SEM images showing a polar view of *Scaevola plumieri* **(A)** and *Scaevola taccada* **(B)** pollen grains. Light microscopy images are shown in the insets. Both species have tricolporate microspinulose grains (further characteristics of *Scaevola* pollen are described in Gustafsson et al., 1997). *S. taccada* pollen grains are slightly larger than *S. plumieri*, but otherwise appear similar.

**Figure 5:**
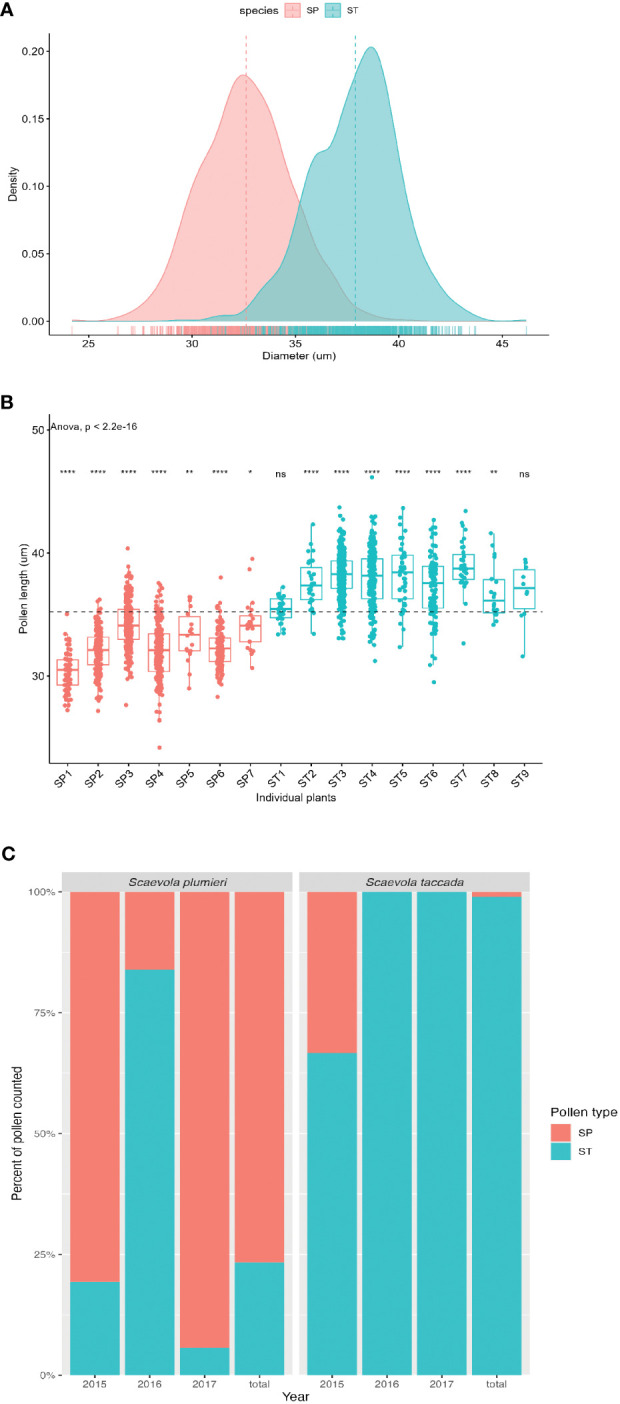
Analysis of pollen size and pollen transfer between *Scaevola* species. **(A)** Frequency distribution of pollen diameters plotted as a density graph from unopened flowers of *S. plumieri* (peach) and *S. taccada* (teal). **(B)** Comparison of pollen sizes from unopened flowers of seven individuals of *S. plumieri* (peach) and nine individuals of *S. taccada* (teal). ANOVA test shows significant differences in pollen sizes from individuals compared to the overall mean (dotted line). ****p ≤ 0.0001; **p ≤ 0.01; *p ≤ 0.05; ns = not significantly different. Overall difference (ANOVA) between species is significant (p < 2.2 x10^-16^) **(C)** Identity of pollen grains found on open-pollinated flowers of each species per year, expressed as a percentage of total pollen grains assigned. Based on pollen size distributions shown in **(A, B)** pollen grains > 37.73 µm were assigned to *S. taccada* (ST; teal) and pollen grains < 32.95 µm were assigned to *S. plumieri* (SP; peach). Plots produced in R (R Core Team, 2023).

Pollen dissected from unopened flowers of *Scaevola taccada* and *S. plumieri* was compared using light microscopy to characterize differences such that each species’ pollen could be identified from samples taken from open-pollinated flowers and pollinators. Morphologically, the two species have nearly identical spherical tricolporate pollen. FE-SEM image analysis (**Figure 4**) illustrates the similarity in pollen surface features in these species. The two species’ pollen can be distinguished based of size (**Figures 5A, B**). The average diameter of *S. taccada* grains was 37.9 ± 2.1 µm (range 29.5 - 46.2 µm) whereas *S. plumieri* grains were on average smaller in diameter at 32.8 (± 2.2) µm (range 24.2 – 40.2 µm).

Analysis of pollen size class distributions collected from open-pollinated flowers from both species demonstrated that pollen transfer was occurring between both species (**Figure 5C**). We analyzed a total of 937 pollen grains removed from *S. plumieri* flowers (from 5 individuals over 3 years), and 376 pollen grains removed from *S. taccada* flowers (from 4 individuals over 3 years). Because the size class distribution of pollen diameters of the two species overlapped, we identified pollen with diameters >37.73 µm as *S. taccada* and pollen with diameters <32.95 µm as *S. plumieri*. We avoided assigning identity to any pollen grains in the size range where the species’ distributions overlapped (~32-37 µm). When we applied this rule to open-pollinated flowers collected in 2017, we found that 5.7% of the identified pollen (264 pollen grains) found on *S. plumieri* indusia belonged to *S. taccada*, while none of the identified pollen found on *S. taccada* indusia (237 pollen grains) belonged to *S. plumieri* (**Figure 5C**). This asymmetry in pollen movement was also observed in 2015 and 2016. Combining three years of data (1313 pollen grains measured; 800 with assigned identity), *S. taccada* flowers had only 1% *S. plumieri*-assigned pollen on their indusia, whereas *S. plumieri* had 23.4% *S. taccada*-assigned pollen on their indusia (**Figure 5C**). Although we analyzed similar numbers of open-pollinated flowers from each species (~15 flowers), the likelihood of encountering an invasive flower with native pollen deposited by a pollinator is much lower than the reverse.

### Insect pollen analyses

We analyzed pollen found on the bodies of Hymenopteran visitors. Not all visitors were observed or captured in every year, preventing year to year comparisons for individual insect species, however; nearly all captured visitors carried pollen from both *Scaevola* species (**Table 2**). In addition, we found variation in the amount of *Scaevola* pollen carried by these visitors, depending on which plant species they had most recently visited. For example, pollen found on *Apis mellifera* bees had sizes of pollen consistent with having visited both species, and higher amounts of pollen from the species it was captured on. This pattern was observed in all but three individual insects. Our analyses confirm that bees and wasps are capable of transferring pollen between both species of *Scaevola*. We found much less pollen on the bodies of visiting wasps (*Campsomeris, Polistes, Stictia*) suggesting that these species are less effective pollinators for *Scaevola* compared to bees.

**Table 2:** Identity of pollen carried by visiting insects.

Pollinator (plant last visited) x number individuals analyzed	Percentage of pollen carried (%)	
	*S. plumieri pollen* (<32.95 µm)	*S. taccada pollen* (>37.73 µm)
*Apis mellifera* (SP) x 2	98.8	1.20
*Apis mellifera* (ST) x 3	39.6	60.3
*Centris decolorata* (SP) x 1	50.0	50.0
*Centris decolorata* (ST) x 4	16.7	83.3
*Campsomeris trifaciata* (SP) x 4	99.8	0.20
*Lasioglossum* sp. (SP) x 1	69.6	30.4
*Megachile concinna* (SP) x 1	40.0	60.0
*Prionyx thomae* (SP) x 3	87.2	12.8
*Stictia signata* (SP) x 1	83.3	16.7
*Xylocopa mordax* (SP) x 1	34.8	65.2
*Xylocopa mordax* (ST) x 1	5.60	94.4

Potential pollinators were captured on either *Scaevola plumieri* (SP) or *Scaevola taccada* (ST) and their pollen loads were analyzed and categorized as either SP (<32.95 um) or ST (>37.73 um). Pollen grains falling in between these sizes was not counted as part of the total. Pollinators were collected over three years (2015-2017).

### Nectar volume and sugar concentration

In all three years (2015-2017), we measured no significant differences in the average volume per flower or the average percent sugar in nectar between *S. taccada* and *S. plumieri* in any one year (**Table 3**). There were, however, significant differences in both volume and sugar concentration among different years of collection, suggesting abiotic influences on both. *Scaevola plumieri* had significantly higher nectar volumes in 2016 as compared to 2015 (p < 0.001) and 2017 (p = 0.01). *S. taccada* also had significantly higher nectar volumes in 2016 as compared to 2015 (p = 0.0003) and lower volumes in 2015 as compared to 2017 (p = 0.03). Sugar content was significantly lower for *S. plumieri* in 2016 as compared to 2015 (p = 0.04) but not different from 2017. For *S. taccada*, 2016 sugar content was significantly lower than 2017 (p = 0.04) but not significantly different than 2015. When all three years of observation were combined, *S. taccada* had significantly higher nectar volume (p=0.02) than *S. plumieri.*

**Table 3:** Average nectar volume and sugar percentage per flower in *Scaevola* over three years (standard deviation in parentheses).

Average per flower	2015		2016		2017	
	*S. taccada*	*S. plumieri*	*S. taccada*	*S. plumieri*	*S. taccada*	*S. plumieri*
Nectar volume (µL)	0.57 ( ± 0.42)	0.41 ( ± 0.22)	*1.26 ( ± 0.56)	*1.31( ± 0.39)	0.98 ( ± 0.43)	0.39 ( ± 0.37)
Sugar percentage (%)	52.4 ( ± 25.3)	71.0 ( ± 30.1)	§37.3 ( ± 14.4)	†40.2 ( ± 8.1)	57.2 ( ± 5.1)	49.50 ( ± 5.6)

*Nectar volumes of both species were significantly different in 2016 as compared to 2015 and 2017 (p < 0.05) but did not significantly differ between *Scaevola* species in any one year. Sugar concentrations between the two species were also not significantly different in any one year. †Sugar concentration in *S. plumieri* in 2016 was significantly lower than in 2015 (p = 0.041) but not different from 2017. §Sugar concentration in *S. taccada* was significantly lower in 2016 than in 2017 (p = 0.041) but not different from 2015.

### Amine group containing constituents

AGCCs were found in all nectar samples analyzed and we detected a total of 17 different AGCCs in our samples. *Scaevola plumieri* samples were found to contain between 11 and 15 AGCCs while *S. taccada* samples were found to contain 11 to 16 AGCCs. Five of the AGCCs could be identified as the amino acids serine, arginine, threonine, alanine, and proline and were present in both species at varying concentrations. The rest of the amine group containing constituents were unidentified.

#### Intraspecific variation

Conspecific nectar samples were highly variable in total concentration of AGCCs, in concentration of individual AGCCs, and in AGCC composition. The mean correlation coefficient for *S. plumieri* was 0.725 and for *S. taccada* was 0.363, reflecting the variation in both concentration and composition of AGCCs samples of the same species. To compare the variation in concentration and composition separately, we calculated the coefficient of variability as described in Gardener and Gillman (2001). These calculations indicate high variability for both measures, but lower variability in composition. Average coefficients of variability in concentration were 0.94 for *S. plumieri* and 1.14 for *S. taccada*. The same calculation for composition was 0.76 for *S. plumieri* and 1.04 for *S. taccada*.

#### Interspecific variation in concentration of constituents

All but one of the 17 AGCCs detected in our samples were common to both species; AGCC 2 was detected only in *S. taccada*. Despite large sample to sample variation, we detected three AGCCs whose absolute concentrations were significantly higher in *S. taccada* than in *S. plumieri*: proline was measured at 10.52% in *S. taccada* vs. 3.02% in *S. plumieri* (p = 0.037); AGCC 3 at 1.71% in *S. taccada* vs. 0.77% in *S. plumieri* (p = 0.027); and AGCC 8 at 13.04% in *S. taccada* vs. 2.95% in *S. plumieri* (p = 0.015).

#### Interspecific variation in composition of constituents

**Figure 6:**
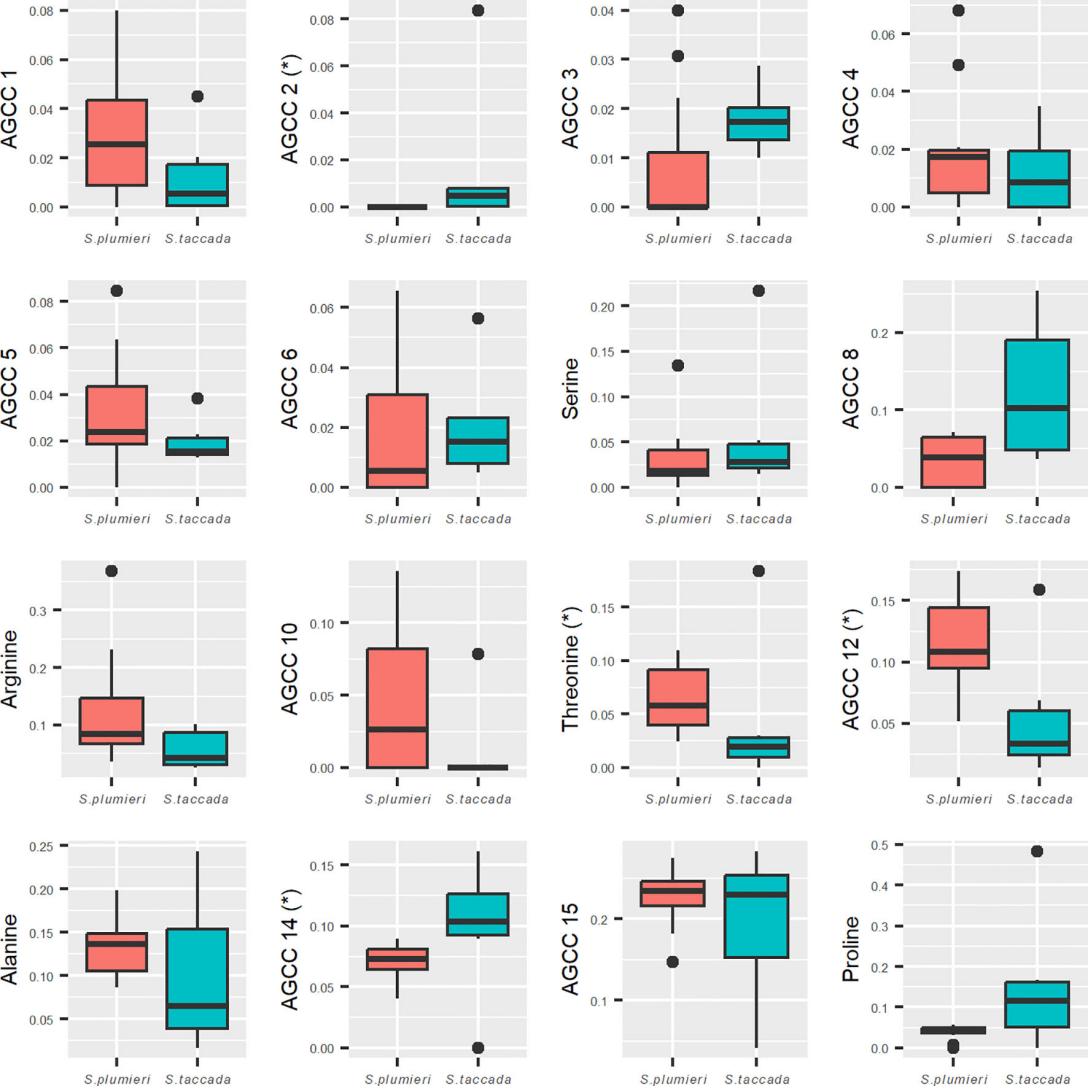
Interspecific variation in composition of amine group containing constituents (AGCCs). Mini-plots compare proportions of 16 different AGCCs between *S. plumieri* (peach) and *S. taccada* (teal). Four constituents (AGCC 2, 12, 14 and threonine) had significantly different proportions in the total AGCC content between the two species. *S. taccada* has proportionally higher amounts of unidentified AGCCs 2 and 14 whereas threonine and AGCC 12 are proportionally higher in *S. plumieri.* Constituent 2 was not detected in *S. plumieri*. Norleucine was used as the internal standard and could not be distinguished from native norleucine and is therefore not included here. Y-axis values represent proportions of each constituent to the total AGCCs present (mass of AGCC/total mass of all ACGGs).

Analyzing the proportions of each AGCC to each other allowed us to make additional distinctions in nectar profiles between the two species that were not apparent when comparing absolute concentrations (**Figure 6**). Threonine was shown to make up a significantly higher proportion of the AGCCs in *S. plumieri* than *S. taccada* (p = 0.037). AGCC 12 was also found to be higher proportionally in *S. plumieri* (p = 0.020). Two unidentified AGCCs (2 and 14) made up a significantly higher proportion of AGCCs in *S. taccada* (both p = 0.027).

## Discussion

### Characterization of floral visitation

Previous studies have observed that flowers of *Scaevola* species are visited predominantly by Hymenopterans and less frequently by Lepidopterans and nectivorous birds (Carlquist, 1969; Ellmore, 2008; Aluri et al., 2019). We also observed primarily Hymenopteran visitors to both native and invasive *Scaevola* at our study sites in Puerto Rico. Bees made up the greatest percentage of visitors in 2016 to both *Scaevola* species, but generally we observed that wasps were more frequent visitors to native *S. plumieri* than to the invasive *S. taccada* (**Table 1**). Year to year variability in visiting species is likely due to environmental factors that affect the availability of other food sources for visiting Hymenopterans and due to our limited time frames of observation in each year.

Because *S. plumieri* and *S. taccada* share the same insect visitors, pollinator competition by the invasive *S. taccada* might lower the visitation rates for the native *S. plumieri*. Alternatively, the abundance of flowers present on *S. taccada* plants (~100s to 1000s depending on plant size) compared to *S. plumieri* plants (~10 to 50) might offer an attractive resource for pollinators thereby facilitating pollinator visits to native. The average visitation rates we observed at the Playa Grande (invaded) site did not differ significantly between the two species, which suggests that *S. taccada* does not monopolize the available pollinators but may ensure similar visitation rates for *S. plumieri* though we have no specific evidence for pollinator facilitation.

Observations at Camuy enabled us to compare visitation rates at a large uninvaded population of *S. plumieri* with the invaded *S. plumieri* population at Playa Grande. At Camuy, we observed visitation rates similar to those at Playa Grande. *Scaevola plumieri* is abundant at Camuy, providing a large floral resource similar to that found at Playa Grande. Therefore, the abundance of the resource, rather than the species, may be the more important factor influencing visitation frequency for *S. plumieri*.

### Analysis of pollen movement

We observed that bees (*Xylocopa, Centris, Apis, Megachile*) carry *Scaevola* pollen on their bodies and in greater quantities compared to visiting wasps (*Campsomeris, Polistes, Stictia*) suggesting that bees are the more effective pollinators for both species of *Scaevola*. This observation is similar to that of Aluri et al. (2019) who found *S. taccada* pollen on the bodies of visiting wasp species (*Campsomeris, Scolia, Vespa*), but in smaller quantities than bees. We captured bees and wasps that carried pollen belonging to both species, which supports our observations that *Scaevola* species at our study site share pollinators. In cases where only one species’ pollen was identified on an insect, it was typically pollen from the species upon which the insect was captured.

Analysis of open-pollinated flowers revealed asymmetrical heterospecific pollen transfer between *Scaevola* species (**Figure 5C**) at Playa Grande. The asymmetry in pollen deposition may be explained by the disproportionately large number of *S. taccada* flowers at this study site. The invasive offers significantly more flowers (100s-1000s) compared to the native (~10-50) and therefore most pollinators are visiting the invasive only, or are visiting the native after visiting the invasive.

Secondary pollen presentation in *Scaevola* could influence measurement of pollen movement. The floral indusium in a *Scaevola* flower initially contains only its own pollen until pollinators remove that pollen and deposit foreign pollen. Thus, newly-opened flowers contain more of its own pollen and older flowers containing more insect-deposited pollen. If our pollen counts were influenced by secondary pollen presentation, we would have seen more homospecific pollen deposition, yet despite this influence, we saw the opposite.

Heterospecific pollen deposition on the native *S. plumieri* could potentially cause stigma clogging, inhibit fertilization, or lead to the formation of native-invasive hybrids (Streher et al., 2020). We are aware of no reports that *S. taccada* can successfully hybridize with *S. plumieri* (Koontz et al., 1996) and efforts to achieve interspecific hybridization in the genus appear to be hampered by embryo failure after fertilization (Luo, 2005). *S. taccada* and *S. plumieri* are not closely related species (Howarth et al., 2003), and thus it would not be expected that they would form natural hybrids. Heterospecific pollen deposition in *S. plumieri* may limit reproductive success by stigma clogging (preventing conspecific pollen tubes from fertilizing embryos) or by yielding non-viable embryos. The effect of heterospecific pollen deposition needs to be further investigated by comparing native seed set and viability at invaded locations versus uninvaded locations.

### Nectar rewards

We observed no significant differences between *S. taccada* and *S. plumieri* in the average nectar volume per flower and average percent sugar concentration; therefore, the influence of these factors on pollinator preference between the invasive and the native appears to be negligible. High variability in these parameters among the collection years suggests that nectar volume and sugar content are influenced by abiotic factors, such as recent rainfall.

Amine group containing constituents of nectar (AGCCs) from each species were compared using measures of absolute concentration and relative abundance (composition). Absolute AGCC concentrations were highly variable, making comparisons between the two species difficult. We therefore also compared the relative abundance (composition) of AGCCs which exhibited less variability.

Identifiable AGCCs that differed between *S. plumieri* and *S. taccada* included proline and threonine. Proline concentration was more than three times higher in the nectar of *S. taccada* than *S. plumieri* (though not significantly different proportionally). Proline is the most abundant amino acid in many angiosperm nectars (Gardener and Gillman, 2002) and has been identified as a specific attractant to honeybees (Carter et al., 2006; Bertazzini et al., 2010; Nepi et al., 2012), which may increase foraging time and result in higher plant fitness. Proline also can be rapidly metabolized, providing a burst of energy that is used for the initial, energy-intensive lift-off phase of insect flight (Carter et al., 2006; Stec et al., 2021). The higher proline concentration in *S. taccada* (10.52%) compared to *S. plumieri* (3.02% in) may give *S. taccada* a pollination advantage over *S. plumieri*.

Threonine was observed to be present at a significantly higher proportion in *S. plumieri* than *S. taccada* and is considered to be an essential amino acid for pollinators (Nicolson and Thornburg, 2007). The impact of threonine on pollinator behavior or preference is not well studied, although microbial contamination of nectar has been observed to decrease threonine concentration (Lenaerts et al., 2016), and so this difference may not be meaningful. Serine, arginine and alanine were not significantly different in concentration or composition, suggesting that these amino acids may be involved in maintaining pollinator relationships for both *S. plumieri* and *S. taccada.* Differences in other, unidentified AGCCs were detected in either concentration or composition (**Figure 6**), but their ecological relevance is unclear without further characterization.

## Conclusions

Our analysis of insect visitation frequencies for native vs. invasive *Scaevola* in Puerto Rico suggest that where these two species co-occur, the introduced *S. taccada* does not monopolize pollinators. The proximity of the invasive may encourage similar insect visitation rates to the native species, despite a much less prolific floral resource offered by the native, though we have no specific evidence to support facilitation by the invasive. Large native-only stands experience similar visitation rates as invaded localities, suggesting that the size of the floral resource may be a more important factor influencing visitation rate than the species composition of the resource.

Nectar rewards do not differ significantly between the two species in terms of volume or sugar content, however the invasive contains a higher concentration of proline which is known to enhance pollinator attraction. Differences in nectar composition may aid in the success of *S. taccada* as an invasive species, however; such differences were not reflected in insect visitation rates we observed.

Our results therefore suggest that a larger threat to the native *S. plumieri* may be habitat encroachment by the introduced species rather than by pollinator monopolization*. Scaevola taccada* is a more effective disperser and colonizer and is much faster growing than the native (Guppy, 1917; Knevel and Lubke, 2004; CABI, 2023). Efforts to protect *S. plumieri* and other native strand species may be best accomplished by increasing public awareness, and by preventing the importation, establishment, and spread of *S. taccada* in these locations as has been proposed in the Cayman Islands (DaCosta-Cottam et al., 2009).

## Data availability statement

The original contributions presented in the study are included in the article/supplementary materials, further inquiries can be directed to the corresponding author/s.

## Author contributions

SS: Writing – original draft, Writing – review & editing, Investigation, Formal analysis, Visualization. AG: Writing – review & editing, Investigation. CP-M: Writing – review & editing, Investigation. NC: Writing – review & editing, Investigation. AW: Writing – review & editing, Investigation. NR: Writing – review & editing. JC: Writing – review & editing, Formal analysis, Visualization. PM: Writing – review & editing, Investigation, Formal analysis, Visualization.
